# Clinical relevance of glucose metrics during the early brain injury period after aneurysmal subarachnoid hemorrhage: An opportunity for continuous glucose monitoring

**DOI:** 10.3389/fneur.2022.977307

**Published:** 2022-09-12

**Authors:** Daniel Santana, Alejandra Mosteiro, Leire Pedrosa, Laura Llull, Ramón Torné, Sergi Amaro

**Affiliations:** ^1^Comprehensive Stroke Center, Institute of Neuroscience, Hospital Clinic of Barcelona, Barcelona, Spain; ^2^Neurosurgery Department, Institute of Neuroscience, Hospital Clinic of Barcelona, Barcelona, Spain; ^3^Institut d'Investigacions Biomèdiques Agustí Pi i Sunyer (IDIBAPS), Barcelona, Spain; ^4^Department of Medicine, Faculty of Medicine and Health Sciences, University of Barcelona, Barcelona, Spain

**Keywords:** subarachnoid hemorrhage, early brain injury, continuous glucose monitoring, stress hyperglycemia, glycemic variability, glucose profile

## Abstract

Hyperglycaemia, hypoglycaemia and higher glucose variability during the Early Brain Injury (EBI) period of aneurysmal subarachnoid hemorrhage (aSAH) have been associated with poor clinical outcome. However, it is unclear whether these associations are due to direct glucose-driven injury or if hyperglycaemia simply acts as a marker of initial severity. Actually, strict glucose control with intensive insulin therapy has not been demonstrated as an effective strategy for improving clinical outcomes after aSAH. Currently published studies describing an association between hyperglycaemia and prognosis in aSAH patients have been based on isolated glucose measurements and did not incorporate comprehensive dynamic evaluations, such as those derived from subcutaneous continuous glucose monitoring devices (CMG). Arguably, a more accurate knowledge on glycaemic patterns during the acute phase of aSAH could increase our understanding of the relevance of glycaemia as a prognostic factor in this disease as well as to underpin its contribution to secondary focal and diffuse brain injury. Herein, we have summarized the available evidence on the diagnostic and prognostic relevance of glucose metrics during the acute phase of cerebrovascular diseases, focusing in the EBI period after aSAH. Overall, obtaining a more precise scope of acute longitudinal glucose profiles could eventually be useful for improving glucose management protocols in the setting of acute aSAH and to advance toward a more personalized management of aSAH patients during the EBI phase.

## Introduction

Hyperglycaemia occurs in about three out of four aSAH patients within the first 72 h after the initial bleeding and it has been associated with poor clinical outcomes and secondary brain injury (1–7). However, it remains to be clarified whether these observed associations are due to direct deleterious effects induced by high blood sugar or if hperglycaemia is simply a marker of stroke severity. Although hyperglycemia has been related to worse outcome in several pathologies, there is conflicting evidence about the benefits of intensive glucose management for improving clinical outcomes in different critical settings (8, 9). Thus, the potential clinical benefit of strict control with intensive insulin therapy has not yet been validated by clinical trials (7, 10–13). Of note, intensive management of blood glucose may result in hypoglycaemia, and both hyperglycaemia and hypoglycaemia have been associated with higher risk of complications during the acute phase of aSAH and with poorer clinical recovery at long-term. Hyperglycaemia is, in part, a consequence of an acute stress response to brain injury and may enhance neuroinflammatory mechanisms and increase the risk of ischemic complications such as delayed cerebral ischemia (DCI) (5–7). Conversely, hypoglycaemia is also dangerous for the brain, which relies on glucose for maintaining its function, and low glucose levels may eventually increase secondary brain injury through a myriad of mechanisms that include a poorer tissue tolerance to ischemia (5–7). Besides, acute fluctuations in serum glucose concentrations are common after aSAH and may also result detrimental (1–7, 14–16). In this review, we will summarize the available evidence on the diagnostic and relevance of glucose metrics during the acute phase of cerebrovascular diseases, focusing in the EBI period after aSAH.

## Glycaemic status in acute cerebrovascular illnesses: How to tackle the problem

Glycaemic dysregulation decisively affects vital and functional prognosis of patients across several cerebrovascular diseases, although it is still unclear how to effectively address the glycaemic status in the acute phase of critical diseases such as aSAH. Traditionally, glycaemic evaluation has relied on static parameters, like glycaemia on admission or premorbid glycaemic status including known-diabetes or glycated hemoglobin (HbA1c) on admission. Nonetheless, glycaemia is a rapidly changing parameter due to both disease evolution and acute management-related factors that are only partially understood. Therefore, static markers may be inadequate to address the true relevance of glycaemic status and to guide insulin therapy in the acute setting. To overcome this limitation, several studies addressed glycaemia as a dynamic variable by means of evaluating the glycaemic variability (GV) (17, 18). In this context, subcutaneous Continuous Glucose Monitoring (CGM) devices have emerged as a potential substitute to point of care standards in the inpatient setting. In brief, CGM devices are usually placed on the thigh, abdomen or arm, and measure interstitial fluid glucose generally through an oxidase-peroxidase reaction. That information is sent to an external device, then allowing a remote, non-invasive, high temporal resolution evaluation of the glycaemic status. Common technical limitations of CGM in an ICU setting include biofilm formation, need for calibration, measurement lag and the interaction of drugs such as acetaminophen or vasopressors (19, 20). Initial studies evaluating the feasibility of CGM in the ICU setting have mainly focused on the accuracy of the technique, before assessing its ability to guide treatments. In general, and in spite of the technical limitations cited above, the device accuracy seems to be acceptable and reproducible across different series and acute clinical settings, with no major safety issues. From a logistic and economic point of view, some reports indicate that CGM devices might significantly reduce nurse workload regarding glycaemic control and might be cost-effective (21, 22).

The exhaustive information obtained through CGM includes static measures such as mean, maximum and minimum glucose levels, the amount of time spent above or below a predefined threshold, and also several dynamic features accounting for GV. CGM also allows the identification of different longitudinal glucose profiles, as well as performing complex analyses of glucose homeostasis, which would be not feasible with conventional testing. The main advantages of CGM, compared to conventional finger prick testing, are the ability to comprehensively analyse a multitude of parameters and to obtain their longitudinal trajectory profiles. Reports on CGM in acute cerebrovascular diseases showed similar-to-better detection of dysglycaemic events, mainly hypoglycaemia, compared to the standard of care. Interestingly, some of these events show circadian variability and nocturnal preference (23, 24), being easily overlooked with the conventional scheduled capillary glucose measurements.

However, the information derived from CGM devices should be interpreted carefully, since definitions and thresholds of the novel glycaemic parameters could be complex and heterogeneous. Beyond the extraction of the aforementioned predefined key metrics, the comprehensive analysis of CGM-derived repeated measurements may require sophisticated analytic tools and pre-planned analytical strategies (19, 20). In addition, evidence regarding which subsets of patients would benefit from wearing a CGM device is still scarce (19).

The ability of CGM to guide intensive insulin treatments (IIT) is also a matter of debate. Although the use of CGM has spread in the outpatient setting for certain indications, its utility in the inpatient setting remains to be demonstrated. Thus, interventional clinical trials based on CGM are scarce in comparison with accuracy and safety studies. A recent report in a cardiac ICU demonstrated good patient and caregivers' acceptance, accuracy and reliability of subcutaneous CGM measurements (25). Indeed, CGM could be of potential great value in the management of acute illnesses, especially in patients at risk for high GV and hypoglycaemia, including those affected by vascular brain injury (19). A retrospective analysis of two randomized controlled trials including critically ill patients that compared CGM-driven IIT vs. conventional arterial point of care (POC)-driven IIT showed that CGM driven-IIT did not result in a significantly reduced GV. However, glucose complexity measures calculated with real time CGM data were predictive of the risk of mortality (26). In one of those trials, real-time CGM-driven IIT was associated with a reduced risk of hypoglycaemic events, an observation that was also replicated in a similarly designed trial including postcardiac surgery patients (27, 28). In the same line, a recent trial comparing insulin dosing driven by CGM measurements vs. standard POC glucose testing in a cohort of hospitalized patients receiving IIT or bolus basal therapy showed earlier hypoglycaemia detection in the CGM group (29).

In summary, CGM technology seems safe, accurate and might be cost-effective in the acute inpatient setting, although its clinical value for guiding IIT or for prognostic purposes in patients who need tight glycaemic control, as those affected by acute brain lesions, remains to be demonstrated. Importantly, the benefit of strict glycaemic control with IIT for improving clinical outcomes in aSAH remains to be validated in clinical trials before an eventual implementation of CGM for guiding IIT (7, 10–13).

## Diagnostic and prognostic relevance of static and dynamic glucose metrics in the acute phase of aSAH

Patients with aSAH may suffer focal and diffuse brain lesions in addition to the initial bleeding, a fact that dramatically impacts the functional and cognitive outcomes at long term. According to the moment of appearance, within or after the first 72 h from the initial bleeding, these brain lesions are classified as part of EBI or DCI, respectively (30–33). Traditionally, DCI secondary to vasospasm has been considered one of the main complications related to poor prognosis in this disease, although therapeutic approaches aimed to control angiographic vasospasm have not shown reliable clinical benefits (33, 34). On the other hand, the severity of EBI, both *via* acute ischemic lesions or microstructural diffuse lesions, is associated with an increased risk of systemic complications and with poorer clinical, cognitive and affective outcomes at long-term (35–37). There is growing evidence of the role that physiopathological changes play in the promotion of EBI after aSAH. These mechanisms include loss of cerebral autoregulation, microthrombosis, enhanced exposure to inflammation and oxidative stress, blood brain barrier (BBB) disruption and apoptotic cell death, among others (38–43).

Hyperglycaemia has been associated with a prothrombotic state, increased neuroinflammation, oxidative stress and BBB disruption in several clinical and preclinical models of central nervous system diseases. A plausible underlying mechanism is the overproduction of mitochondrial reactive oxygen species, which occurs preferentially in tissues that are insulin-independent like the brain (44–49). In experimental SAH, hyperglycaemia promotes neuronal apoptosis and is associated to higher incidence of vasospasm (43, 50). In the vulnerable brain, hyperglycaemia also promotes anaerobic glycolysis leading to the accumulation of lactate, a process thought to contribute to increased brain injury (6, 51–53).

From a clinical standpoint, dysglycaemia is a major prognostic factor in both early and late phases of spontaneous aSAH, as summarized in Table 1. First, hyperglycaemia has been associated with worse clinical and radiological presentation, as well as with higher in-hospital mortality, higher rates of neurological and systemic complications, and ultimately worse short-term and long-term functional outcomes (3, 5, 7, 14, 16, 54–62, 64–66). On the other hand, hypoglycaemia has been strongly linked to in-hospital mortality and incidence of vasospasm (55, 59, 61–63). Otherwise, poor premorbid metabolic control estimated by HbA1c upon admission does not seem to consistently correlate with the acute neurological status nor with DCI or neurological outcome at long-term (64, 65). Some authors have proposed the use of ratios to increase the predictive value of admission blood glucose levels. In this line, the glucose-potassium and glucose-phosphate ratios on admission proved to be a significant predictor of vasospasm, rebleeding, along with acute and mid-term functional outcomes (58, 60, 66, 67).

**Table 1:** Main studies assessing the impact of glucose and brain metabolism in prognosis and complications after spontaneous SAH.

**Author**	**Study type**	**Population**	**Assessment**	**Results**
Barletta et al. (14)	Retrospective	SAH HH >2 in ICU *n* = 42	Mean BG, GV, hypoG	↑GV: ↑incidence of brain infarction ↓GV: No difference in infarction between those with high and low mean BG
Beseoglu et al. (64)	Prospective observational	Non-diabetic aSAH *n* = 78	Admission BG and HbA1c	BG: Correlation with initial neurological status and mF. No correlation with DCI
Eagles et al. (7)	Retrospective	Non-diabetic aSAH *n* = 389	MaxG	MaxG <9, 2 mmol/L correlated with a decreased risk of unfavorable outcome
Helbok et al. (51)	Prospective observational	Poor-grade aSAH *n* = 39	MD glucose, PbtO2, and cerebral perfusion	Admission GCE: ↓brain pyruvate and glucose Non-GCE: ↑brain glucose up to day 7
Jung et al. (58)	Prospective observational	aSAH *n* = 553 cases 553 controls	Admission BG, K^+^	BG: 98.4% hyperG on admission K^+^: 26.0% hypoK^+^, 0.5% hyperK^+^ Severe group: ↑BG and GPR Non-severe group: ↑K^+^
Kruyt et al. (5)	Meta-analysis	aSAH 17 studies (*n* = 3,373) for mean BG 8 studies (*n* = 2,164) for outcome	Weighted mean admission BG	HyperG on admission: increased risk for poor outcome compared with patients without hyperG
Kurtz et al. (15)	Retrospective	aSAH GCS <9 *n* = 28	ICP, PbtO2, MD, GV	↑GV: ↑risk of metabolic distress and hospital mortality
Liu et al. (59)	Retrospective	aSAH in ICU *n* = 1,298	Admission BG	U-shaped relationship between admission BG and 30-days all-cause mortality. Consistent at 90 days
Matano et al. (66)	Retrospective	Surgical aSAH *n* = 333	BG every 4 h, K^+^	GPR, BG and K^+^ correlated with DCI GPR correlated with VSp and poor outcome
McGirt et al. (3)	Retrospective	Surgical aSAH *n* = 97	BG every 6 h	Persistent hyperG: worse outcome at 2 weeks and 10 months Corrected admission hyperG: No correlation Mean daily BG: ↑40% poor outcome per 10mg/dL of increase
McIntyre et al. (61)	Retrospective	aSAH *n* = 217	Admission and hospitalization mean BG, MinG, MaxG, GV, DM history, HbA1c, BMI, insulin use	↑mean hospitalization BG: ↑mortality risk, mortality and complications predictor MinG: correlation with VSp MaxG: correlation with complications Admission BG >140 mg/dL: ↑ mortality DM history, BMI, HbA1c: No correlations
Naidech et al. (55)	Retrospective	aSAH *n* = 172	Mean BG, MaxG, MinG, GV	↑WFNS: ↑maxG, ↑mean BG, ↑GV, ↓inG Brain infarction: ↓minG Symptomatic VSp: ↓minG Worse outcome correlated to ↑admission. BG, ↑maxG, ↑mean BG, ↑maxG, ↓minG
Okazaki et al. (16)	Retrospective	aSAH in ICU *n* = 122	BG every 6 h, GV	↑GV, ↓minG: correlated with poor outcome
Pappacena et al. (57)	Retrospective	Non-diabetic aSAH. (*n* = 6,098) or TBI (*n* = 11,812) in ICU	MaxG, minG, mean BG, GV	↓MaxG, ↓MinG, ↓ mean BG, ↓GV in aSAH and TBI compared to overall ICU population Correlations between dysglycemia and mortality were stronger in aSAH/TBI than in general ICU population
Sadan et al. (69)	Retrospective	aSAH in ICU *n* = 2,451	BG, GV	↓GV correlated with survival in non-diabetic and well-controlled diabetic aSAH patients
Schlenk et al. (63)	Prospective non- randomized	aSAH *n* = 28	BG, MD glucose	MD low and high-glucose episodes occurred independently of BG
Sun et al. (65)	Retrospective	aSAH *n* = 119	Admission glycemic gap (aGG)	↑ aGG predicted mortality and poor outcome better than admission BG, and correlated with DCI and EVD placement
Thiele et al. (62)	Retrospective (impact study)	aSAH *n* = 834	Admission and average BG. Strict glucose control protocol (GCP)	↑verage BG correlated with ↑risk of death ↑admission BG showed no correlations GCP initiation: No effect on in-hospital mortality. ↑hypoG. ↑ VSp
van Donkelaar et al. (56)	Retrospective	aSAH in ICU *n* = 285	BG and lactate first 24 h	↑BG correlated with DCI ↑lactate correlated with poor outcome
Wang et al. (60)	Retrospective	Non-diabetic aSAH *n* = 744	Admission BG and GPR	↑GPR correlated with clinical and radiological severity and predicted rebleeding and poor outcome
Zetterling et al. (71)	Retrospective	aSAH with EVD *n* = 19	BG every 3 h. MD glucose. MD/BG ratios	Weak positive correlation between BG and MD glucose. Insulin therapy correlated with ↓MD although BG remained normal
Zhang et al. (67)	Retrospective	aSAH *n* = 198	Admission GPR	Positive correlation between GPR and WFNS ↑GPR in patients with poor outcome

aSAH: aneurysmal Subarachnoid Hemorrhage; HH, Hunt and Hess scale; BG, Blood Glucose; GV, Glucose variability; HypoG, Hypoglycemia; mF, Modified Fisher scale; DCI, Delayed Cerebral Ischemia; MaxG, Maximum glucose; MD, Microdyalisis; PbtO2, Brain tissue Oxygen tension; GCE, Global Cerebral Edema; HypoK, Hypokalemia; HyperK, Hyperkalemia; HyperG, Hyperglycemia; GPR, Glucose/Potassium Ratio; GCS, Glasgow Coma Scale; ICP, Intracranial Pressure; MinG, Minimum Glucose; VSp, Vasospasm; DM, Diabetes Mellitus; BMI, Body Mass Index; WFNS, World Federation of Neurosurgical Societies scale; TBI, Traumatic Brain Injury; EVD, External Ventricular Drain.

Even when hyperglycaemia on admission is a very sensible biomarker, it has been suggested that GV is a more reliable tool to predict outcomes (3, 14–16, 68, 69). Several formulas have been proposed to express the GV, yet they usually rely on comparison of low temporal resolution assessments of capillary glucose. A remarkable observation in terms of GV implications is the differential behavior between diabetic and non-diabetic patients during the acute phase of aSAH. Prior studies have suggested that the association between GV and outcome occurs in non-diabetic and well-controlled diabetic patients, but not in those with poorly controlled diabetes (69). Therefore, tolerance to high or low glucose levels may vary according to the premorbid glycaemic status.

Importantly, blood glucose levels might not always reflect those present in the brain interstitium as assessed with mycrodyalisis catheters (70, 71). In acute aSAH, glucose transport through the BBB is impaired, causing that even normal levels of blood glucose result relatively insufficient to supply the raised brain metabolic demand that occurs during the EBI period (71). This phenomenon poses patients with aSAH at a vulnerable state, where glycaemic fluctuations may provoke brain metabolic distress and secondary injury. Remarkably, systemic GV even within normal limits of blood glucose levels has also been associated with the incidence of cerebral metabolic distress, evaluated though multimodal monitoring with cerebral microdialysis probes (15, 51). Henceforth, GV instead of absolute glucose levels might better reflect the clinical course of severe aSAH, and eventually guide glycaemic-control interventions.

To our knowledge no studies regarding the prognostic relevance of glucose temporal profiles have used GCM devices in the specific setting of aSAH. Indeed, there is no sufficient evidence to conclude that longitudinal evaluation of glycaemic patterns have a greater prognostic value during the EBI period of aSAH. The vast majority of published data on hyperglycaemia and prognosis in aSAH patients are based on scheduled capillary glucose measurements as implemented in standard clinical practice (e.g., capillary glucose determinations every 6 h within the first days after bleeding) and have not considered including comprehensive dynamic evaluations, such as those derived from CGM devices. Arguably, using CGM to obtain accurate glycaemic profiles during the acute phase of aSAH could help define the relevance of glycaemia as a prognostic factor in this disease, as well as underpin its contribution to secondary focal and diffuse brain injury.

## Diagnostic and prognostic yield of glucose monitoring in acute cerebrovascular diseases

Studies focused on hyperacute glucose monitoring in cerebrovascular diseases have included mainly patients affected by acute ischemic stroke (AIS). In this setting, both admission and acute-phase hyperglycaemia have been associated with mid-term and long-term morbidity and mortality following a J-shaped fashion, as well as with higher incidence of infarct growth, ischemic recurrence risk and post-stroke cognitive impairment (72–76). After alteplase infusion, hyperglycaemia correlates with worse recanalization status, higher odds of symptomatic haemorrhagic transformation (sICH) and poorer clinical recovery (77–80). Higher GV after alteplase administration has also been associated with higher concentrations of circulating markers of inflammation and with worse clinical outcomes (81). In the same line, after mechanical thrombectomy, glycaemic disarrangements have been associated with worse outcomes, higher frequency of sICH and higher risk of death, although no link was found with recanalization rates (82–85). In intracerebral hemorrhage (ICH), hyperglycaemia at admission has been related to worse functional outcomes and death, in both diabetic and non-diabetic populations (86, 87). Reassuringly, a recent meta-analysis that evaluated the prognostic relevance of GV in both AIS and ICH showed a significant association between lower GV and reduced mortality at 30 and 90 days after stroke (88).

Nonetheless, studies using CGM in stroke are still scarce and have been based in modest population sizes. Disglycaemia measured by CGM is frequent after acute cerebrovascular events and has been linked to infarct growth in AIS (89, 90) and with poor prognosis after AIS and ICH (91), as shown in Table 2. It is noteworthy that, in these populations, CGM devices significantly improved hypoglycemia detection compared to standard clinical practice, thus opening a window of opportunity for CGM in patients that require aggressive glycaemic control (23, 24). However, to date, intensive glucose lowering in the acute phase of AIS has not reliably proven a clinical benefit, pointing the lack of consensus on hyperglycaemia definition or blood glucose targets (92, 93).

**Table 2:** Studies assessing potential clinical impact of CGM in cerebrovascular acute disease management.

**Author**	**CGM manufacturer**	**Population**	**Outcome**	**Findings**
Ribo et al. (89)	Minimed, medtronic	MCA stroke receiving IV alteplase (*n* = 47)	Infarct growth. Short term clinical course	Hyperglycemia during OT associated with poorer outcome and greater infarct growth in DWI
Shimoyama et al. (90)	Minimed, medtronic	ICA/MCA stroke (*n* = 78)	Infarct growth	Mean glucose & AUC >140 associated with infarct growth at 24 h and 72 h
Wada et al. (91)	iPro 2, medtronic	AIS (*n* = 58) and AHS (*n* = 42)	mRs ≥3 at 3 months	Mean glucose, AUC 8 & distribution time >8 were linked to mRs ≥ 3
Nukui et al. (23)	Freestyle libre pro, abbott	AIS within 7 days (*n* = 39)	Hyper and hypoglycemia detection	Glucose events after AIS are frequent and show circadian variability
Palaiodimou et al. (24)	iPro 2, medtronic	AIS (*n* = 48) and AHS (*n* = 14)	Clinical outcome at 3 months. Hypoglycemia detection	Higher MAG relates to lower likelihood of neurological improvement. Hypoglycaemia is better detected by CGM and is mainly nocturnal

Studies limited to accuracy and feasibility of CGM were excluded. Of note, up to date there are no trials regarding CGM in the setting of aSAH. MCA, Middle Cerebral Artery; OT, Occlusion Time; DWI, Diffusion-Weighted Imaging; ICA, Internal Carotid Artery; AUC, Area Under the Curve; AIS, Acute Ischemic Stroke; AHS, Acute Haemorrhagic Stroke; mRs, Modified Rankin Scale; MAG, Mean Absolute Glucose.

## Conclusions and future directions

Hyperglycaemia, hypoglycemia and glucose variability during the acute phase of aSAH have been associated with an increased risk of in hospital complications and with poor long-term functional recovery. However, it is unclear whether these associations are due to direct deleterious effects induced by glucose dysregulation or if hyperglycaemia is an epiphenomenon related to the initial bleeding severity. In addition, it remains inconclusive whether there are specific longitudinal glycaemic profiles during the EBI period with special prognostic implications and/or more prone to lead to secondary brain injury. Arguably, the use of comprehensive dynamic evaluations, such as those derived from CGM devices, could aid to obtain a more precise understanding of such a highly dynamic process that is conditioned by both systemic complications and specific therapeutic approaches. A more accurate knowledge on glycaemic patterns during the acute phase of aSAH could increase our understanding of the relevance of glycaemia as a prognostic factor in this disease and to underpin its contribution to secondary focal and diffuse brain injury. In this context, the evaluation of biomarkers related with brain microvascular, metabolic and microstructural integrity using quantitative advanced MRI along with the quantification of circulating and cerebrospinal fluid (CSF) molecules related with brain injury could be highly informative for identifying glucose profiles associated with higher brain damage after aSAH. More research is needed to understand the most appropriate and informative timing for glucose monitoring, and how to combine glucose metrics with surrogate markers of acute brain injury. Eventually this information may lead to improve glucose management protocols in the setting of acute aSAH, to optimize the design of clinical trials aimed to modulate glucose levels and to advance toward a more personalized management of aSAH sufferers during the EBI period.

## Author contributions

DS, AM, LP, LL, RT, and SA participated in writing the manuscript and editing critical parts of this review. LL, RT, and SA gave the final approval for this submission. All authors contributed to the article and approved the submitted version.

## Funding

We thank the Spanish Ministry of Economy and Competitiveness for the grant given to SA and RT [project PI19/00936 funded by Instituto de Salud Carlos III and co-funded by the European Regional Development Fund (ERDF)], the Fundació la Marató de TV3 for the grant given to SA (grant number 17/C/2017). DS is supported by a grant from Hospital Clinic de Barcelona (Contracte Clínic de Recerca Emili Letang-Josep Font).

## Conflict of interest

The authors declare that the research was conducted in the absence of any commercial or financial relationships that could be construed as a potential conflict of interest.

## Publisher's note

All claims expressed in this article are solely those of the authors and do not necessarily represent those of their affiliated organizations, or those of the publisher, the editors and the reviewers. Any product that may be evaluated in this article, or claim that may be made by its manufacturer, is not guaranteed or endorsed by the publisher.
